# A Comparative Study of Choledochoduodenostomy Versus Open Choledochotomy With T Tube Placement in a Hospital Lacking Endoscopic Retrograde Cholangiopancreatography

**DOI:** 10.7759/cureus.71438

**Published:** 2024-10-14

**Authors:** Farrukh Hassan, Kumar Gaurav, Krishan Kumar, Kamlesh Kumar, Praveenkumar A, Venkatesh N, Shreya, Swaroop Sanat Sahu, Sameer Kumar Mahto, Balamurali B

**Affiliations:** 1 General Surgery, Rajendra Institute of Medical Sciences, Ranchi, IND; 2 Community Medicine, Rajendra Institute of Medical Sciences, Ranchi, IND

**Keywords:** choledochoduodenostomy, gall bladder diseases and gallstones, prolonged length of hospital stay, recurrent choledocholithiasis, surgical obstructive jaundice, t tube

## Abstract

Background: Choledocholithiasis, or stones in the common bile duct (CBD), has two types: primary stones that form in the CBD and secondary stones that migrate from the gallbladder. Management includes endoscopic, laparoscopic, and open surgical methods. In India, the availability of endoscopic retrograde cholangiopancreatography (ERCP) and laparoscopic surgery is limited often necessitating open procedures. Commonly, open choledochotomy followed by T tube placement was performed. However, postoperative management/management of retained stones can be challenging, requiring referrals for ERCP or revision surgery. This study aims to compare the outcomes of choledochoduodenostomy versus the T tube approach in a hospital setup where ERCP is either unavailable or cumbersome.

Materials and methods: This was a retrospective comparative study carried out at Rajendra Institute of Medical Sciences (RIMS), Ranchi, Jharkhand, India. The study was approved by the Institutional Ethics Committee of RIMS, Ranchi. A total of 62 patients who underwent operations for choledocholithiasis (CBD dilation ≥ 1.2 cm) from January 2023 to January 2024 in the Department of General Surgery of RIMS, Ranchi, were analyzed.

Results: The mean age group was 52 years ± 11.5 years, and two-thirds were females with a male-to-female ratio of 1:1.8. The most common presentation was biliary colic (87 %), followed by jaundice (45%). Around three-fourths of them had multiple calculi (n = 46). A total of 36 patients underwent T tube (58.1%) and 26 underwent choledochodudoenostomy (41.9 %). The mean operating time was higher for the T tube approach but not statistically significant. The hospital stay for the patients was significantly higher for the T tube approach with a p-value of <0.001. The mean T tube in situ duration was 17.60 days ± 1.2 days. On performing a T tube cholangiogram postoperatively, it was observed around one-fourth of them had T tube filling defect (n = 8), signifying the residual stone presence and referral to higher center due to unavailability of ERCP. The incidence of wound infection was significantly high among the patient who underwent T tube with a p-value of 0.017, and postoperative bile leak was significantly high among the patient who underwent T tube with a p-value of 0.047.

Conclusion: Based on our retrospective analysis, we suggest choledochoduodenostomy was safer and more efficient in aspects of lesser operating time, minimal hospital stay, less or nil retained stones, lesser postoperative wound infection, bile leakage, and possessing advantages especially to elderly patients both economically and psychologically.

## Introduction

Choledocholithiasis, the presence of stones in the common bile duct (CBD), can be categorized into two types based on their origin: primary and secondary. The latter, which is more common, originates in the gallbladder and migrates into the CBD. In contrast, primary stones develop within the CBD itself. The classical clinical presentation of choledocholithiasis includes biliary colic, jaundice, cholangitis, and pancreatitis. In these cases, intermittent obstruction caused by CBD stones results in fluctuating bilirubin levels, whereas persistent obstruction can lead to cholangitis. This condition is well described by Charcot’s triad (fever, pain, jaundice) or Reynolds’ pentad (Charcot’s triad plus altered mental status and hypotension). Choledocholithiasis is noted in approximately 10% to 15% of patients who already present with cholelithiasis, and it is more frequently observed among elderly patients [1]. Various techniques are employed for the management of choledocholithiasis, including endoscopic, laparoscopic, and open surgical approaches. Among these, the endoscopic approach is the most commonly used technique. However, it is not always feasible and is associated with significant morbidity and mortality [2], including complications such as pancreatitis and hemorrhage. Additionally, endoscopic retrograde cholangiopancreatography (ERCP) is relatively expensive and not uniformly available in many healthcare settings. Similarly, the laparoscopic approach requires skilled personnel and expensive equipment [3]. Among open surgical approaches, supraduodenal choledochotomy with T tube placement remains the preferred and standard management technique for choledocholithiasis. However, the T tube approach has several disadvantages, including prolonged hospital stays, bile leakage upon T tube removal, and the potential need for revision surgery. An alternative technique that has proven useful in the management of choledocholithiasis is choledochoduodenostomy [3,4]. This relatively uncommon procedure was first introduced by Riedel in 1888 in Europe, but the first successful operation was carried out in 1891. In benign conditions, the presence of stenosis in the distal portion of the CBD, also known as funnel syndrome, is one of the most common indications for choledochoduodenostomy [2]. Other important indications for biliary bypass operations (choledochoduodeonostomy, choledochojejunostomy, hepaticojejunostomy) include biliary strictures, malignant obstruction of the biliary system due to pancreatic or biliary ductal carcinomas, and cases requiring revision surgery due to residual or recurrent stones, larger impacted calculi in the CBD, or concomitant strictures and bile duct calculi [5]. Choledochoduodenostomy is considered highly curative [3]. But it is not typically the first choice for the management of choledocholithiasis except for the conditions mentioned above. In many hospitals in India, the availability of ERCP and laparoscopic surgery is limited due to various factors. Consequently, these facilities often rely on open surgical approaches for the management of choledocholithiasis. Despite its disadvantages, the traditional open choledochotomy followed by T tube placement is often employed when ERCP is unavailable. However, postoperative management of patients with retained stones remains a concern, as these patients may require referral to other centers for ERCP or revision surgery. This study aims to compare the outcomes of choledochoduodenostomy versus the T tube approach in a hospital wherein ERCP is either unavailable or cumbersome.

## Materials and methods

This was a retrospective comparative study carried out at Rajendra Institute of Medical Sciences (RIMS), Ranchi, Jharkhand, India. The study was approved by the Institutional Ethics Committee of RIMS, Ranchi, with memo no 08/2023. A total of 62 patients who underwent operations for choledocholithiasis (CBD dilation ≥ 1.2cm), those with biliary stricture, and those aged more than 18 years from January 2023 to January 2024 in the Department of General Surgery of RIMS, Ranchi, were analyzed. Patients with CBD dilation < 1.2cm were excluded due to the risk of postoperative anastamotic stricture, incomplete medical records, those lost to follow-up, those with choledochoduodenostomy for malignant conditions, and those with concomitant calculi in CBD with malignancy were also excluded.

Operating method

*T Tube Approach* 

A right upper quadrant incision was given; however, an upper midline incision can be used as well. Gentle palpation of the distal bile duct was done to find the offending stone. Stay sutures are then placed, and choledochotomy is performed in the supraduodenal bile duct. Flushing of the duct with a soft rubber catheter to remove the offending stones was done. With the complete removal of stones, a T tube of size 16-18 Fr was placed.

Choledochoduodenostomy

The duodenum is kocherized widely to allow for tension-free anastomosis, and CBD was dissected completely along its distal anterior surface. A longitudinal duodenotomy was made close to the bile duct along the long axis of the first part of duodenum, perpendicular to the choledochotomy. For a side-to-side anastomosis, 2 cm CBD incision was made along the long axis of the bile duct as close to the duodenum as possible. After performing a CBD exploration and clearing the duct of stones (palpatory method, stone removing forceps and free flow of saline into duodenum), a side-to-side single layered anastomosis was made with absorbable monofilament suture (PDS 3-0) and drain was placed.

Data analysis

The data collected was entered into MS Excel (Microsoft Corporation, Redmond, Washington, United States) and then exported to the data editor of: IBM SPSS Statistics for Windows, Version 27 (Released 2020; IBM Corp., Armonk, New York, United States) for analysis. Continuous variables were expressed as mean ± SD, and unpaired t-test (Mann-Whitney U test in case of nonnormally distributed continuous variables) was the statistical tool used for finding the association. These include age, operating time, number of days t tube placed, and postoperative stay. Categorical variables were summarized as frequencies and percentages. These include age group, gender, clinical features, wound infection, and recurrence. To evaluate the association between categorical variables, chi-square test was used.

## Results

These tables shows that the study population belongs to the age group of 41 to 60 years with a mean age of 52 ± 11.5 years. Around two-thirds of them were females with a male-to-female ratio of 1:1.8 (Table 1).

**Table 1:** Distribution of cases according to demographic variables (n = 62)

Variable	N	%
Age category		
25-40 years	8	12.9
41-60 years	39	62.9
>60 years	15	24.2
Sex*		
Female	40	64.5%
Male	22	35.5%
Other statistics		
Mean	52.26	
Std. deviation	11.485	
*male-to-female ratio 1: 1.8		

**Figure 1:**
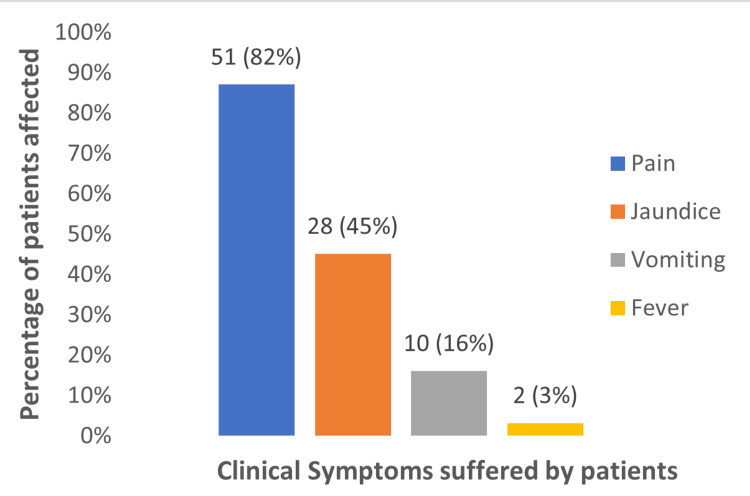
Distribution of the clinical presentation among the patients (n = 62)

The clinical features of the study population have been shown in the above figure showing most of them having biliary colic as the common presentation followed by jaundice as the second most common presentation (Figure 1).

In the study population, around three-fourths of them had multiple calculi in the CBD (n = 46) (Table 2).

**Table 2:** Distribution of multiple and solitary calculi among the cases (n = 62)

Calculi status	N	%
Multiple	46	74.19%
Solitary	16	25.81%

**Figure 2:**
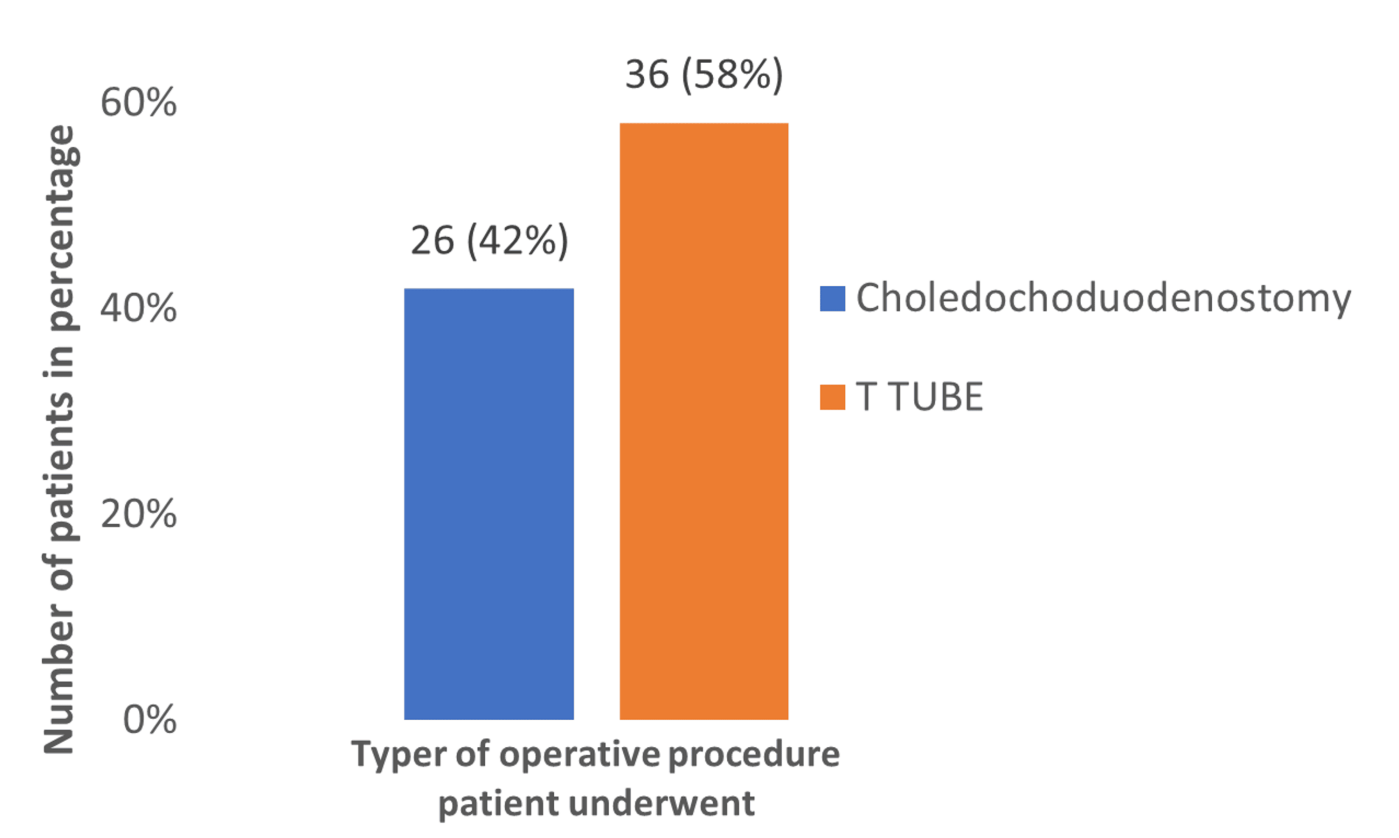
Percentage of operating procedure for choledocholithiasis (n = 62)

In the total study population of 62 patients, most of them underwent T tube approach (58.1 %), showing that the most preferred technique was T tube among surgeons (Figure 2).

**Figure 3:**
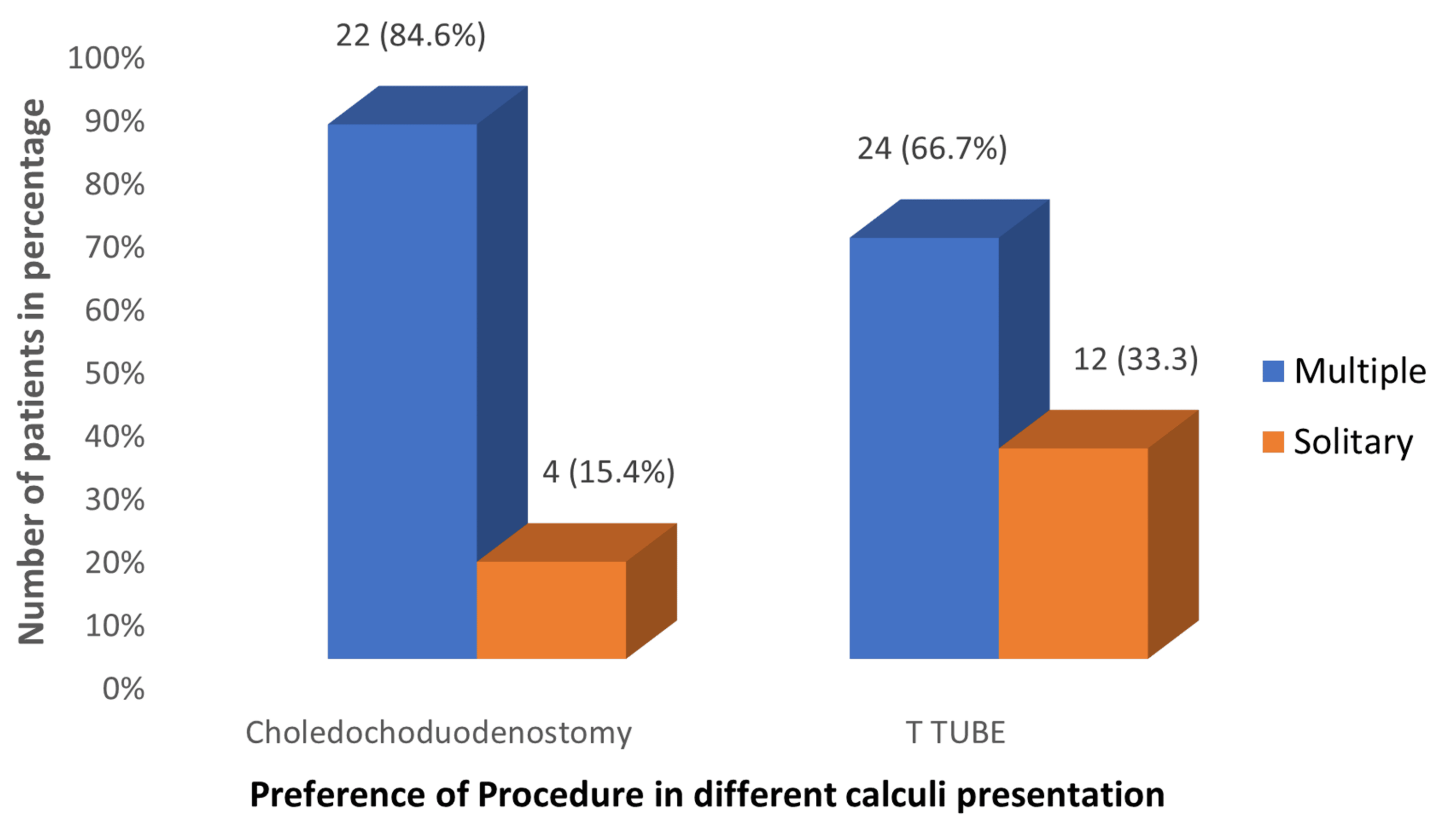
Preference of procedure opted in multiple and solitary calculi cases, respectively (n = 62)

Preference of opting T tube approach was more in the patients who presented with solitary calculi, whereas choledochodudoenostomy was preferred more commonly among the patients with multiple stones. The solitary calculi operated under choledochoduodenostomy were those with impacted and distal calculi (Figure 3).

The operating time is on the higher side in the T tube approach when compared to choledochoduodenostomy, but the result was not statistically significant (Table 3).

**Table 3:** The operating time of both procedures *Unpaired T-test

Operating time in minutes	Choledochoduodenostomy	T tube	p-value*
Mean	130.38	138.50	>0.05
Std. deviation	27.746	36.029	
Range	75	180	
Minimum	90	90	
Maximum	165	270	

The hospital stay for the patients was significantly on the higher side for the patient who underwent T tube approach with p-value < 0.001 (Table 4).

**Table 4:** The hospital stay for the patients in both procedures *Unpaired T test

Hospital stay in days	Choledochoduodenostomy	T tube	p-value*
Mean	9.54	19.56	<0.001
Std. deviation	1.303	3.581	
Range	4	16	
Minimum	8	14	
Maximum	12	30	

Among the patients of the T tube approach, it was observed that the T tube was in situ for a mean duration of 17.60 ± 1.2 days (Table 5).

**Table 5:** The number of days of the T tube in situ among the cases (n = 36) *Among 36 T tube cases, six cases were missing due to referral

T tube in situ time*	
Mean	17.60
Std. deviation	1.276

On performing postoperative cholangiogram for patients who underwent T tube approach (n = 32), it was observed that around 25% (n = 8) of them had T tube filling defect signifying the residual stone presence. Hence, these patients were referred to a higher center for ERCP as there was no availability of ERCP in our hospital (Table 6).

**Table 6:** The percentage of residual stone after open choledochotomy with T tube placement (n = 32)

T tube filling defect	N = 32	%
No	24	75%
Yes	8	25%

Wound infection was considerably high among the patients who underwent T tube approach, which is statistically significant with p-value = 0.017 (Table 7).

**Table 7:** The percentage of postop wound infection in both procedures, respectively (n = 62) *Chi-square test

Postop wound infection	Choledochoduodenostomy		T tube		p-value*
	N	%	N	%	0.017
Absent	24	92.3%	24	66.6%	
Present	2	7.7%	12	33.3%	

Postoperative bile leak was considerably high among the patients who underwent T tube approach, which is statistically significant with p-value = 0.047 (Table 8).

**Table 8:** The postop bile leak in both procedures, respectively (n = 62) *Chi-square test

Postop bile leak	Choledochoduodenostomy		T tube		p-value*
	N	%	N	%	0.047
Absent	26	100%	31	86%	
Present	0	0%	5	14%	

Among the choledochoduodenostomy patients, on follow-up ,it was observed that one-fifth of them had alkaline reflux gastritis. None of the patients who underwent T tube procedure reported alkaline reflux gastritis (Table 9).

**Table 9:** The percentage of alkaline reflux gastritis among operated patients (n = 62)

Alkaline reflux gastritis	Choledochoduodenostomy		T tube	
	N	%	N	%
Absent	21	80.77%	36	100%
Present	5	19.23%	0	0%

## Discussion

From our study, on comparing the two groups, the mean hospital stay for T tube patients was 19.56 ± 3.5 days, whereas choledochoduodenostomy patients had a mean hospital stay of 9.5 ± 1.3 days. The longer hospital stay observed in T tube patients is due to the need for T tube cholangiography on the 8th to 10th postoperative day to ensure no retained stones, to the fact that they are at high risk of developing nosocomial infection during the longer hospital stay. These findings are consistent with a study done by Keighley et al., a prospective study, that observed a mean hospital stay of 14 days for T tube patients and 6.8 days for choledochoduodenostomy patients [6]. In contrast, a long-term prospective study by Mihmanli et al., which followed up with patients for five years, revealed that the T tube approach was superior to choledochoduodenostomy, due to the higher incidence of alkaline reflux gastritis in choledochoduodenostomy, with a p-value of less than 0.05 [7]. However, in our study, only about one-fifth of the patients (n = 5) reported symptoms of alkaline reflux gastritis. During postoperative period, five patients developed features of cholangitis which was managed conservatively. A randomized controlled trial by Lygidakis demonstrated that choledochoduodenostomy has a low morbidity rate of 8.8%, with no mortality and no need for resurgery, and T tube patients had a mortality rate of 4.4% and also required resurgery in 20.9% of cases [8]. Similarly, a case series by Schein et al. indicated that T tube patients had a higher mortality rate compared to choledochoduodenostomy patients, where the latter was observed to be an excellent therapeutic and prophylactic procedure for managing choledocholithiasis and its predicted complications [9]. This is consistent with our study, where approximately 25% of patients who underwent the T tube approach had a filling defect on T tube cholangiography, necessitating revision surgery in this population. Additionally, the wound infection rate in our study was 33.33% among T tube patients postoperatively, compared to only 7.7% among choledochoduodenostomy patients. These results align with those of Keighley et al. who reported that 38 out of 116 patients experienced postoperative wound infections, with a higher rate observed in T tube patients compared to those who underwent choledochoduodenostomy and primary closure [6]. This highlights the fact that T tube patients are more prone to wound infections due to various factors, such as longer hospital stays and leakage during T tube removal. A study by Stewart et al. [10] states that longer hospital stays are associated with a higher incidence of nosocomial infections, with the overall mean hospital stay of patients with infections being 3.5 times longer than those without infections. In line with our findings on the efficacy of choledochoduodenostomy, studies by Malik et al. and Aggarwal et al. have emphasized the efficacy and safety of choledochoduodenostomy, provided that suturing techniques are meticulous and precise [11,12]. Furthermore, Hoerr et al. stated that choledochoduodenostomy is a simple and less time-consuming procedure [13]. In our study, we compared the operating time between the T tube and choledochoduodenostomy approaches, finding that the mean operating time for the T tube was 138.50 ± 36 minutes, while for choledochoduodenostomy, it was 130.38 ± 27.7 minutes. Although the difference was not statistically significant, the operating time was slightly longer for the T tube approach.

Limitations

The main drawback of the study is that it was done as a retrospective study, hence giving rise to recall bias. Also, there are considerable number of cases that could not be included because of improper documentation and the lack of data needed for the study like the status of the patients with retained stones who were referred outside. Although infrequent [1], sump syndrome and anastomotic site stricture couldn’t be studied owing to the lack of longer follow-up in our study design. Follow-up till two years postoperatively, every six months once with liver function test (LFT), upper gastrointestinal endoscopy (UGIE), ultrasonography (USG) whole abdomen, is needed. Prospective study would have resolved the above shortcoming and would have been helpful in an elaborative study of the same. The hospital where this study was done was lacking ERCP facility which was the prime reason for the loss of follow-up in patients who were referred outside for ERCP. Hence, the outcome in these group of T tube patients was not studied in an elaborate manner. Due to the small sample size, the result of the study cannot be generalized for the whole country; hence, this could have been better studied with a larger population in a prospective study.

## Conclusions

In our retrospective study, we observed that the T tube group had statistically significant early postop morbidities, also possessing the burden over the patients due to longer stay. Although the study had limitations, this was the first study to describe the comparison between choledochoduodenostomy and T tube approach in a hospital setup lacking ERCP and found that choledochoduodenostomy was more safe and efficient in aspects of lesser morbidity (retained stones, operating time, hospital stay, wound infection, bile leakage) and possessing advantages, especially to elderly patients both economically and psychologically. We recommend that a randomized controlled trial provide more scientific evidence to determine the efficiency of choledochoduodenostomy and open choledochotomy with T tube placement techniques.
